# Principal component tests: applied to temporal gene expression data

**DOI:** 10.1186/1471-2105-10-S1-S26

**Published:** 2009-01-30

**Authors:** Wensheng Zhang, Hong-Bin Fang, Jiuzhou Song

**Affiliations:** 1 Department of Animal and Avian Science, University of Maryland, College Park, MD 20742, USA; 2 Division of Biostatistics, University of Maryland Greenebaum Cancer Center, Baltimore, MD 21201, USA

## Abstract

**Background:**

Clustering analysis is a common statistical tool for knowledge discovery. It is mainly conducted when a project still is in the exploratory phase without any priori hypotheses. However, the statistical significance testing between the clusters can be meaningful in helping the researchers to assess if the classification results from implementing a clustering algorithm need to be improved, even after the cluster number has been determined by a well-established criterion. This is important when we want to identify highly-specific patterns through classification.

**Results:**

We proposed to use a principal component (PC) test, which is an implementation of an exact *F *statistic for the measures at multiple endpoints based on elliptical distribution theory, to assess the statistical significance between clusters. A challenge in the implementation is the choice of the number (q) of principal components to be considered, which can severely influence the statistical power of the method. We optimized the determination via validation according to a permutation test based on the clustering to be evaluated. The method was applied to a public dataset in classifying genes according to their temporal gene expression profiles.

**Conclusion:**

The results demonstrated that the PC testing were useful for determining the optimal number of clusters.

## Background

Data clustering is a common technique for statistical data analysis used in many fields [1], including machine learning, data mining, pattern recognition, and image analysis. Theoretically, clustering analysis identifies and classifies objects (or individuals) based on the similarity of the characteristics they possess. It seeks to minimize within-group variation and maximize between-group variation and results in a number of heterogeneous groups with homogeneous contents. The general categories of clustering methods include tree clustering (hierarchical clustering), block clustering, *k*-means clustering, and model-based clustering [1]. The evaluation of clustering analysis is a critical challenge in both theory and application.

The performance of clustering analysis can be assessed statistically in order to determine the appropriate clustering methods and cluster number [2]. Pseudo F statistic [3] is widely used for partitioning clustering algorithms, such as k-means, and has been included in the procedure FASTCLUST of SAS software [4]. BIC (Bayesian information criterion) is a well-established statistic based on standard statistical theory and fits model-based clustering procedures [5], which has been widely applied in bioinformatics [6-9]. Silhouette score [10] provides a measure of how well a data point was classified when it was assigned to a cluster according to both the tightness of the clusters and the separation between them. It has been used together with PAM (Partitioning Around Medoids) clustering algorithm [1]. Recently, the so-called Gap statistic was proposed [11], which can use the output of any clustering algorithms for the optimization of cluster number. Furthermore, clustering algorithms are commonly assessed from other angles, such as robustness, stability, consistency, and functional congruence of the members of the same cluster [2,12-18].

On the other hand, while clustering analysis is mainly conducted when we are still in the exploratory phase of our research and do not have any prior hypotheses, the statistical significance testing between the clusters can be meaningful. The testing can help us to assess whether the classification results from running a clustering algorithm need to be improved, even after the cluster number has been determined by a well-established criterion. This is important in the clustering of genes on the basis of the temporal expression profiles. In order to extract specific knowledge about gene function from the expression profiles [19-21], researchers usually hope to have the number of clusters as large as possible but the contrasts between the clusters, each of which corresponds to a co-regulation pattern, should be statistically significant in general.

The significance testing between the clusters can be done by using Hotelling's *T*^2^, the multivariate counterpart of Student's-*t *[22]. But when the number of measurement points is large and the size of samples is relatively small, the results from Hotelling's test are usually unstable [23]. Using the invariance of elliptical distribution theory, a type of exact *t *and *F *tests was proposed [23], which can be applied to high-dimension data with a small size of samples. The tests are based on the sum aggregates of original variables similar to O'Brien's method [24] but superior to the latter in maintaining the prescribed level of significance. Two direct implementations of the method are a one-fold principal component (PC) test corresponding to the exact *t *test and a multi-fold principal component test corresponding to the exact *F *test. The comparison of PC test and *T*^2 ^clearly demonstrated the fact that the stabilizing effect of principal components and PC test made better use of the factor structure of the data of multiple end-points.

Microarray technology allows thousands of genes to be measured simultaneously on a single slide. Unsupervised learning on the basis of clustering analysis of microarray temporal gene expression data has been widely studied in order to discover classes of expression patterns and identify groups of genes that are regulated in a similar manner [7,13,19,20]. However in literature the evaluation of clustering analysis was limited to the global assessment of clustering methods. In this paper, we proposed to use principal component tests based on the exact *F *test for multiple endpoint measures [23] to assess the significance of the contrasts between the gene clusters from different clustering algorithms and implemented it on a public data set. The testing can be conducted after the global evaluation for improving clustering analysis.

## Results

### Clustering and patterns

**Figure 1:**
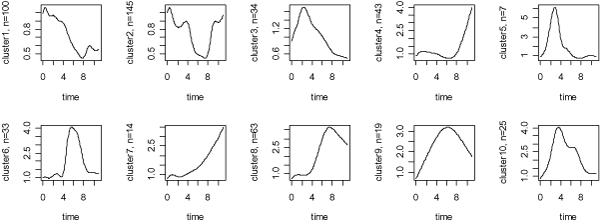
**Expression patterns of genes for the ten clusters from Iyer el al. (1999)**. The time points 1–12 correspond to 15 min, 30 min, 1, 2, 4, 6, 8, 12, 16, 20, and 24 hours) after the serum stimulation.

The first clustering (CL1) to be evaluated was published by Iyer et al [25]. It was obtained by using an agglomerative hierarchical clustering algorithm as mentioned above and contained ten clusters with sizes ranking from 7 to 145. We modeled each cluster by using the smoothing splines technique with the knot number equal to 12 and the patterns are shown in Figure 1. The curves were smoother than the profiles in Iyer et al. [25], where the averages from the measures of the member genes for each cluster were used for graphical purposes.

**Figure 2:**
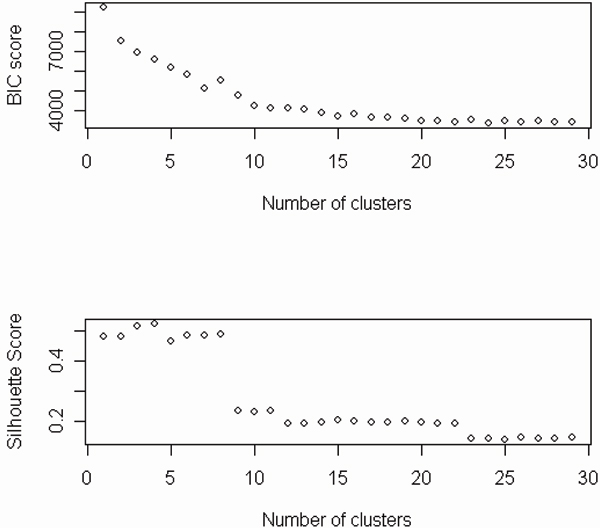
BIC and Silhouette score plots for implementing SSClust and PAM, respectively.

**Figure 3:**
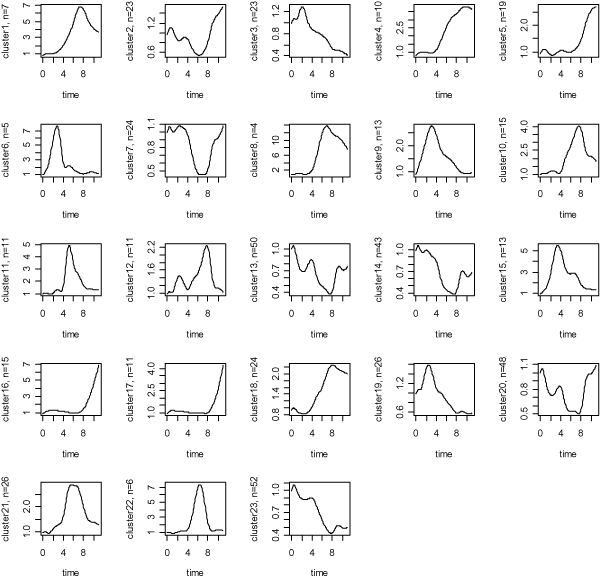
**Expression patterns of genes for the 23 clusters with size over 2 obtained by using SSClust**. The time points 1–12 corresponding to 15 min, 30 min, 1, 2, 4, 6, 8, 12, 16, 20, and 24 hours after serum stimulation.

We obtained the second clustering (CL2) by using a model-based clustering method (SSClust) with BIC criterion, in which the 483 genes (probes) were divided into 25 clusters with the sizes ranking from 2 to 52. In following analysis, the 3 cluster with 2 genes were not considered. It should be noted that the BIC plot did not have the expected "U" shape in this application (Figure 2). Therefore, the determination of the number of clusters was based on local minima of the score. The patterns of the clusters from SSClust are demonstrated in Figure 3. The use of Partitioning Around Medoids (PAM) with silhouette score criterion (Figure 2) the third clustering (CL3) which contained 5 clusters with 303, 35, 43, 96, and 6 genes respectively.

A major difference between CL1 and CL2 was that the two big clusters in the former were divided into two or more smaller classes in the latter. For example, the aggregate of the cluster1 and cluster2 in CL1 approximately corresponded to the aggregate of the cluster2, cluster7, cluster13, cluster14, cluster20 and cluster23 in CL2. In CL3, the major (62%) 483 gens were classified into the first cluster, which largely corresponded to the first biggest cluster in CL2. But the expression patterns of genes of these clusters were very different. Therefore, implementation of PAM with silhouette score criterion does not seem fit for the addressed dataset.

### Determination of q value

**Figure 4:**
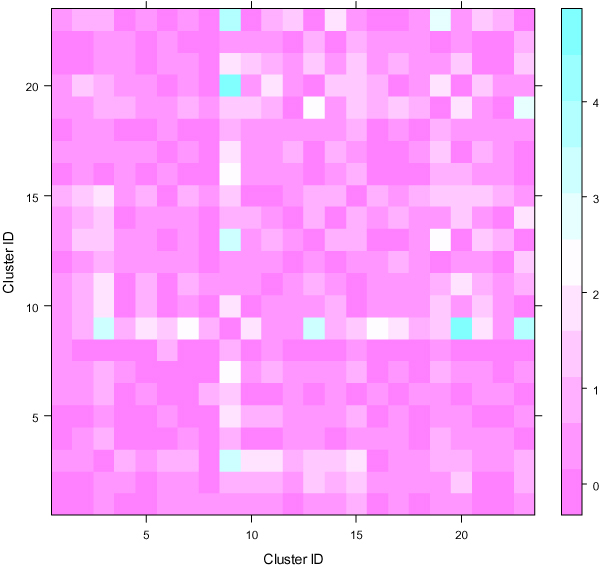
**A set of permutation tests with the number (q) of principal components considered equal to 2 on the clustering (CL2) obtained by using SSClust**. Each color-coded square represents negative logarithm (with 10 as the base) of the p-value for the corresponding cluster contrast.

Using the permutation method described in the Methods section, the number of principal components to be considered, was chosen as q = 2 for all the three applications. Figure 4 shows the results of a set of permutation tests with this parameter setting on the clustering (CL2) obtained by implementing SSClust, in which the proportion of the contrasts with p-value smaller than 0.05 was approximately equal to this value. Our previous simulation study showed that the proportion was far lower than 0.05 when q = 1 and could be larger than 0.1 when q ≥ 3 in the cases with small sample size. Therefore, this choice kept a balance between controlling the type I error and having high statistical power.

### Statistical evaluation of clustering

**Figure 5:**
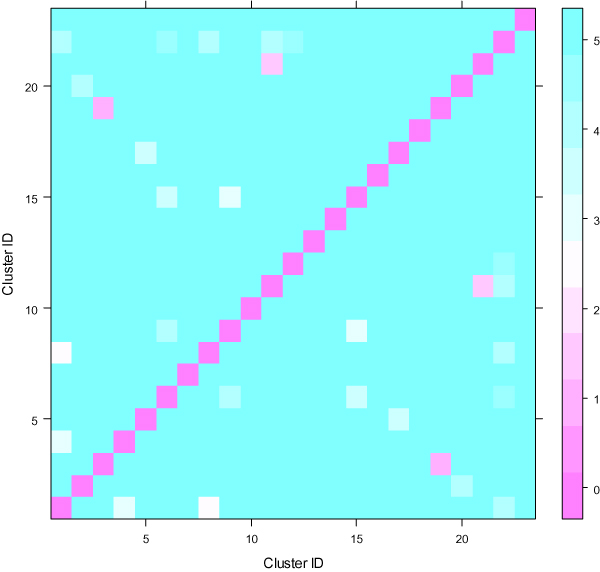
**Two-fold principal component tests for CL2 obtained by using SSClust**. Each color-coded square represents negative logarithm (with 10 as the base) of the p-value for the corresponding cluster contrast.

Two-fold PC tests showed that all the contrasts between the clusters in CL1 were extremely significant (p < 0.01). For CL2, except for two contrasts (cluster11 versus cluster21 and cluster3 versus cluser19) which had p-value larger than 0.1, all other contrasts were statistically significant (p < 0.01) (Figure 5). As mentioned in Section 3.1, the combination of the cluster1 and cluster2 in CL1 was approximately divided into 6 smaller classes in CL2. The statistical significance between them demonstrated that the clustering in Iyer et al. [25] was inadequate for identifying distinguishable gene expression patterns over the time process. The case for CL3 was completely different from CL1 and CL2. Except for the contrasts between the fourth and fifth clusters and between the second and third clusters, all other contrasts were not significant (p > 0.05). This provided support for the conclusion in the last section about the applicability of the PAM method to the addressed data set.

### Biological evaluation of clustering

As mentioned above, the two big clusters in CL1 approximately corresponded to six smaller clusters in CL2, but all the contrasts between these clusters were statistically significant (p < 0.01). The question is whether more biological knowledge about the division of CL1 can be obtained from the division of CL2. The biologically functional enrichment analysis (Table 1) of the gene lists of eight clusters showed that the finer division (compared with CL1) in CL2 represented more specific relationships between the clustering and biological function. For example, three clusters in CL2 (CL2-2, CL2-14, and CL2-20) shared four of the ten PANTHER biological processes [26] which were enriched in the genes contained in the cluster2 in CL1 (CL1-2). A drawback of the finer division was that some small clusters, such as CL2-7 and CL2-13 could not be mapped to any biological function.

**Table 1:** Biologically functional enrichment analysis of the gene lists of eight clusters ^a^

Cluster ID	Enriched PANTHER biological Processes	p-value ^b^
CL1-1	lipid, fatty acid and steroid metabolism	
	(GO: 0006629)	0.0003
	steroid metabolism (GO: 0008202)	0.0006
	cholesterol metabolism (GO: 0008203)	0.0253
		
CL1-2	blood clotting (GO: 0007596)	0.0068
	cell cycle (GO: 0007049)	0.0079
	oncogenesis	0.0101
	nucleoside, nucleotide and nucleic acid metabolism (GO: 0006139)	0.0119
	embryogenesis (GO: 0009700)	0.0435
	cell cycle control (GO: 0000074)	0.0376
	mRNA transcription (GO: 0006366)	0.0825
	cell proliferation and differentiation	0.0843
	(GO: 0031054; GO: 0008283) cell structure and motility (GO: 0007010)	0.0867
	immunity and defense (GO: 0006952)	0.0953
		
CL2-2	cell cycle	0.0325
		
CL2-7	No	
		
CL2-13	No	
		
CL2-14	immunity and defense (GO: 0006952)	0.0313
	developmental processes (GO: 0007275)	0.0432
	oncogenesis	0.0926
		
CL2-20	nucleoside, nucleotide and nucleic acid metabolism (GO: 0006139)	0.0796
		
CL2-23	lipid, fatty acid and steroid metabolism (GO: 0006629)	0.0084
	steroid metabolism (GO: 0008202)	0.0560

^a ^The aggregate of CL-1 and CL-2 approximately corresponds to the combination of CL2-2, CL-7, CL-13, CL-14, CL-20 and CL-23.

^b ^Corrected with Bonferroni method for multiple testing.

## Discussion

Clustering analysis is a widely used tool for knowledge discovery. Moreover, it is applied as a routine method in biology in the post-genomic era. The evaluation of clustering is a problem in its application. In this study, we compared the results of different clustering algorithms from a unique angle by testing the statistical significance of the contrasts between the clusters. In our knowledge, this paper is the first investigation of this kind. We used q-fold PC test which is an implementation of Lauter's exact *F *tests [23] for the measures of multiple endpoints. The method is superior to Hotelling's *T*^2 ^[22] because of the stabilizing effects of the principal components, especially for the data with small sample size. This is important when we want to identify highly-specific patterns via clustering analysis.

The significance of the proposed clustering evaluation includes three aspects. Firstly, the results can tell us if the clustering is meaningful, at least from a statistical standpoint. A good clustering algorithm should meet a basic criterion, i.e., the clusters should be statistically distinguishable. In other words, all of the contrasts between the clusters should be statistically significant at a certain confidence level. Second, it can be helpful in the determination of cluster numbers. For example, in the analysis of temporal gene expression data mentioned above, both the BIC plot did not have the expected "U" shape. Thus, the determination based on a local minimum value may be equivocal and questionable. The results of the PC tests demonstrated that dividing the 483 genes (probes) into 18–20 clusters is appropriate. Finally, the method is extremely useful for the improvement of the results from a clustering analysis by demonstrating which clusters can be combined because of the lack of significant difference between them.

The number (q) of principal components to be considered is a challenge for the PC test. We optimized the determination via validation according to permutation test based on the clustering to be evaluated. In this way, the choice of q is determined by the data and clustering methods. It is superior to the choice based on cumulative energy content (CEC) because the latter needs an artificial threshold of the CEC percentage. More importantly, from the permutation test, we can assess the validity of the PC test itself in controlling type I error.

An alternative approach to the evaluation of clustering of genes based on the temporal expression profiling is biological validation. In this paper, we conducted biologically functional enrichment analysis of the gene lists of several clusters of interest. The results showed that the finer division of clusters from SSClust, a model-based clustering algorithm, can provide more specific relationships between clusters and biological functions.

It is worthy to note that the information from the biological validation is usually limited because the temporal gene expression profiles of the genes involved in a biological process can be very diverse, including, for instance, inverse co-regulation or co-regulation with a time lag or a combination of both [21,27].

## Conclusion

The proposed PCA test method was applied to a public dataset in classifying genes according to their temporal gene expression profiles. The results demonstrated that the PC testing were useful for determining the optimal number of clusters. We also anticipate that the method could be used for pattern identification and similarity analysis.

## Methods

### Data

The initial data set, published by Iyer et al. [25], describes the transcription levels of genes detected by 517 gene probes, corresponding to 497 unique genes, during the first 24 h of the serum response in serum-starved human fibroblasts. By using an agglomerative hierarchical clustering method, the authors [25] detected 10 major gene expression profile clusters among the differentially expressed genes of the serum response. The ten classes contained 465 unique genes or 483 gene probes. Our work was focused on the data of these 483 gene probes with the log-transformed expression ratios as the variables. The gene symbols of 239 annotated genes were provided by Lagreid et al (2006).

### Principal component tests

The q-fold principal test (PC) used in this paper is implemented on the basis of a type of *t *or *F *statistic for high-dimension data.

Assume there are n individuals (genes) and from each one we have p observations at different time points. Assume p-dimensional distribution for ***x***_*i*_(i = 1, 2, n), i.e. ***x***_*i *_~ ***N***(**μ**_***i***_, **Σ**). Denote $X=({x^{\prime }}_{1},{x^{\prime }}_{2}\cdots {x^{\prime }}_{n}{)}^{\prime }$, a p × n matrix representing the gene expression. We have

(1) ***X ***~ ***N***_***p ***× ***n ***_(***M***, **Σ **⊗ ***I***_***n***_),

where $M=({\mu^{\prime }}_{1},{\mu^{\prime }}_{2}\cdots {\mu^{\prime }}_{n}{)}^{\prime }$, **Σ **is a variance and covariance matrix.

For assessing if the two groups (clusters) to which the n genes belong are statistically distinguishable, the null hypothesis to be tested is **μ**_**1 **_= **μ**_**2 **_= ... **μ**_***n***_, i.e.

(2) $${\text{H}}_{0}:M=\mu {1^{\prime }}_{n},$$

The deviations from the hypothesis are to be represented by the contrast ***Mk***, where ***k ***is an n-dimensional vector with ***k'k ***= 1 and ${1^{\prime }}_{n}k=0$. Let n^(1) ^and n^(2) ^represent the numbers of genes in the two populations (clusters), respectively, vector ***k ***can be calculated with following equation,

(3) $$k=\sqrt{\frac{{\text{n}}^{\left(1\right)}{\text{n}}^{\left(2\right)}}{{\text{n}}^{\left(1\right)}+{\text{n}}^{\left(2\right)}}}\left(\begin{array}{c} \frac{1}{{\text{n}}^{\left(1\right)}}1_{{\text{n}}^{\left(1\right)}}\\ -\frac{1}{{\text{n}}^{\left(2\right)}}1_{{\text{n}}^{\left(2\right)}} \end{array}\right),$$

Denote $\bar{X}=X1_{n}{1^{\prime }}_{n}/n$ and let ***D ***be a p × q matrix consisting of the first q (1 < q < min(n, p)) eigenvectors of the solution of the following general eigenvalue problem

(4) $$(X-\bar{X})(X-\bar{X}{)}^{\prime }D=diag(X-\bar{X})(X-\bar{X}{)}^{\prime })D\Lambda ,$$

where **Λ **is the q × q diagonal matrix of q largest eigenvalues, then, ***Z ***= ***D'X ***has a matrix elliptical contoured distribution [28]. Based on the invariance of elliptically contoured distributions, if H_0 _holds, the statistic

(5) $$F=\frac{\text{n}-\text{q}-1}{\text{q}}k^{\prime }Z^{\prime }G^{-1}Zk,$$

exactly follows F distribution with q and n-q-1 as the degrees of freedom [23],

where $G=Z\left(I_{n}-\frac{1}{n}1_{n}{1^{\prime }}_{n}-kk^{\prime }\right)Z$. For a given n and p, the power of this statistic is dependent on the choice of q. When q = 1, the statistic (5) has t-distribution with degree of freedom n-2.

### Determination of q value

The number (q) of principal components to be considered is a challenge for the q-fold PC test. A solution is the choice based on cumulative energy content (CEC). However, the threshold of the CEC percentage has to be artificially determined. Here, we developed a permutation test based on the clustering to be evaluated. Let I_c _be a vector containing the cluster IDs of the genes in the clustering. By shuffling, we get another vector ${\text{I}}_{\text{c}}^{*}$ which has all elements of I_c _arranged in a random order. We, then, replace I_c _with ${\text{I}}_{\text{c}}^{*}$ and carry our significance testing on the $\text{M}=\frac{1}{2}\text{k}\times (\text{k}-1)$ contrasts (k is the cluster numbers) between clusters using PC test with different q (q = 1, 2,...). For each q, we count the number (m) of the random contrasts with p-value smaller than the prescribed error of type-I at α, such as 0.05, and calculate the ratio R = m/M. Finally, we chose the minimum q which, R approximately equals to α. If the cluster number is small, the shuffling-testing procedure should be repeated several times.

### Clustering methods

The results from three clustering algorithms were evaluated in this paper. Following is a simple description of these methods.

#### Agglomerative hierarchical clustering

An agglomerative hierarchical clustering procedure produces a series of partitions of the data, P_n_, P_n-1_,..., P_1_, the first P_n _consisting of n single object "clusters", the last P_1_, consisting of a single group containing all n cases. At each stage the method joins together two clusters which are closest together (most similar) [19]. Differences between methods in this category arise because of the different ways of defining distance (or similarity) between clusters.

#### Model-based clustering with smoothing splines (SSClust)

A model-based method is based on fitting a statistical model (a mixture of Gaussian distributions) to the data [5]. Generally, a cluster membership (or membership probabilities) of a gene is regarded as an unknown parameter(s) which is estimated along with other distributional parameters via the method of maximum likelihood. In the case of temporal gene expression data, the means of the Gaussian distributions are defined with a set of curves which can be solved using spline techniques [6,7,29]. In this paper, we used Ma et al's procedure (SSClust) which is based on smoothing splines [7,30]. BIC was used to determine the optimal numbers of clusters. It is calculated as

(6) $$\text{BIC}=-2\log_{10}(\text{L})+(\sum_{\text{i}=1}^{\text{k}}{\text{v}}_{\text{k}}+4\text{k})\log(\text{N})$$

where L is the likelihood for the mixture model, N is total gene number, k is the cluster number, and v_i _is the numbers of free parameters for i^th ^cluster which is equivalent to the sum of the trace of the smoothing matrix [30]. A small BIC score indicates strong evidence for the corresponding clustering.

#### Partitioning Around Medoids (PAM)

PAM is a generalization of the well-known *k*-means algorithm. It operates on the dissimilarity matrix of the given data set [1]. Compared with the ordinary *k*-means, PAM is more robust, because it minimizes a sum of dissimilarities instead of a sum of squared Euclidean distances. PAM first computes *k *representative objects, called medoids. A medoid can be defined as a characteristic a cluster, whose average dissimilarity to all the objects in the cluster is minimal. After finding the set of medoids, each object of the data set is assigned to the nearest medoid. That is, object *i *is put into cluster *v*_*i*_, when medoid *mv*_*i *_is nearer than any other medoid *m*_*w*_. We used the *pam *program in R package "*cluster" *in Bioconductor, where the optimal number of clusters is selected on the silhouette plot. Silhouette score [10] is obtained by taking the mean of the average silhouette width for all clusters and silhouette width is defined as

(7) $$\text{S}(\text{i})=\frac{\text{b}(\text{i})-\text{a}(\text{i})}{\max(\text{a}(\text{i}),\text{b}(\text{i}))}$$

where *a*(*i*) is the average distance of gene *i *to other genes in the same cluster, *b*(*i*) is the average distance of gene *i *to genes in its nearest neighboring cluster. Like BIC, a small silhouette score indicates evidence for the corresponding clustering.

### Functional enrichment analysis

A web-tool in PANTHER classification system [26] was used for the biologically functional enrichment analysis by comparing the lists of member genes contained in each cluster of interest with gene from H. Sapiens in NCBI. Only PANTHER biological processes, most of which can be exactly mapped to a Gene Ontology (GO) term [31], were investigated at detail. The p-values were firstly calculated on the basis of hyper-geometric distribution theory followed by correction for multiple testing using the Bonferroni method. Because the correction method is conservative, in the following text a biological process with adjusted p-value < 0.1 was considered as "significant".

## Competing interests

The authors declare that they have no competing interests.

## Authors' contributions

WZ and FH carried out statistical analysis and prepared manuscript. JS supervised the analysis and writing. All authors contributed to the design of the project. All authors read and approved the final manuscript.
